# Salivary cortisol is associated with cognitive changes in patients with fibromyalgia

**DOI:** 10.1038/s41598-020-79349-0

**Published:** 2021-01-14

**Authors:** Yi-Ju Lin, Yu-Chieh Ko, Lok-Hi Chow, Fu-Jung Hsiao, Hung-Yu Liu, Pei-Ning Wang, Wei-Ta Chen

**Affiliations:** 1 Division of General Neurology, Department of Neurology, Taipei Veterans General Hospital, No. 201, Sec. 2 Shih-Pai Rd, Taipei, 112 Taiwan; 2 School of Medicine, National Yang-Ming University, Taipei, Taiwan; 3 Department of Ophthalmology, Taipei Veterans General Hospital, Taipei, Taiwan; 4 Department of Anesthesiology, Taipei Veterans General Hospital, Taipei, Taiwan; 5 Brain Research Center, National Yang-Ming University, Taipei, Taiwan

**Keywords:** Neuroscience, Neurology

## Abstract

Fibromyalgia (FM) is a stress-related chronic pain disorder with common cognitive complaints. This study characterized cognitive dysfunction in patients with FM and explored whether these changes are linked to altered cortisol levels. Consecutive 44 patients with FM and 48 healthy controls were enrolled for the assessments of subjective and objective cognitive functions and diurnal levels of salivary cortisol (sampled at awakening, 30 min after awakening, 3 pm, and bedtime). All measurements were compared between the groups and evaluated for clinical correlation. The FM group had more subjective cognitive complaints and performed poorer in objective cognitive testing in memory (delayed recall in Chinese Version Verbal Learning Test and Taylor Complex Figure Test), language (Boston Naming Test), and executive domains (Wisconsin Card Sorting Test) after adjustments for education. The diurnal cortisol levels of patients with FM tended to be lower, especially at 30 min after awakening and bedtime. Moreover, moderate positive correlations existed between the Chinese Version Verbal Learning Test, Boston Naming Test and the morning cortisol levels within the FM group. We suggested the altered cognitive function in FM may be linked to stress maladaptation. Future studies are warranted to elucidate whether stress management improves cognitive performance in patients with FM.

## Introduction

Fibromyalgia (FM) is a chronic pain disorder characterized by widespread body pain and several associated symptoms such as fatigue, cognitive problems, unrefreshed sleep, and depression^1^. Although a complex interaction between genetic disposition and environmental factors may cause FM, its pathophysiology remains undetermined^2^. Many patients with FM identified stress or a stressful event during their lifetime as a pivotal trigger for their chronic pain. In fact, patients with FM reported a higher proportion of early life adversities such as physical and sexual abuse^3^; moreover, post-traumatic stress syndrome (PTSD) is a well-identified comorbidity of FM^4^. The disabling pain per se and complex comorbidities in FM (psychiatric and several others including migraine, irritable bowel syndrome, bladder hyperactivity, and restless leg syndrome) tremendously affect the quality of life of patients^5^. Therefore, FM may be regarded as a stress disorder^2^, and stress may be a window into a thorough understanding of this elusive disorder.

The most studied physiological system of stress is the hypothalamic–pituitary–adrenal axis and its downstream product cortisol^6^. A lower cortisol level has been reported in many stress-related disorders such as PTSD^7^ and chronic fatigue syndrome (CFS). In FM, however, the basal cortisol level and their diurnal change tended to be lower but inconsistent across studies^8–10^, as discussed in a recent meta-analysis^11^. These inconsistent results of cortisol levels in FM might be because of different methods of measurement and the lack of a gold standard^11^. Similar to FM, an animal study^12^ demonstrated chronic stress induced a hypoactive pattern of cortisol through enhanced negative feedback^13^, whereas childhood maltreatment (chronic stress) was associated with higher cortisol levels in patients with FM in the afternoon^14^. Based on the association between hypocortisolism and fatigue experience in CFS, the altered cortisol levels in FM might be considered a maladaptive response to stress^15^. However, some researchers argue that this is a protective mechanism because a low cortisol level may reduce allostatic load or enhance the body’s defense system against chronic inflammation^16^.

Cognitive problems, a core symptom of FM, were reported in more than 76% of patients with FM^2^, majorly involving memory, executive, and language domains^17^. In fact, FM is the only pain syndrome that incorporates cognitive changes into its diagnostic criteria. Cognitive changes are also pivotal for the assessment of functional disability in FM, such as that using the revised Fibromyalgia Impact Questionnaire (FIQR)^18^. In a recent review, decreased mental reserve due to pain, fatigue, and depression has been proposed to cause the cognitive symptoms of FM^17^. Nevertheless, few studies have explored the role of stress in relation to cognitive changes in FM. In animal studies, chronic stress can lead to structural and functional changes in some neural correlates of pain perception or pain modulation, notably in the hippocampus, which may concomitantly affect cognitive functions^2,19^. The hippocampus is vulnerable to stress owing to the high expression of glucocorticoid receptors^20^. In human studies, hypercortisolism is hypothesized to be crucial in the pathogenesis of Alzheimer’s disease^21^, whereas the connection between lower cortisol levels and cognitive dysfunction has been observed in Addison’s disease^22^, PTSD^23^, and the normal population^24^. Although either a low or high cortisol level beyond the physiological range seems detrimental to cognitive function, how cortisol affects cognitive function in FM remains to be investigated.

This study hypothesized that cortisol levels are altered in patients with FM, and this alteration is related to cognitive performance in FM. Accordingly, in the present study, we used comprehensive subjective and objective cognitive assessments to determine whether patients with FM display deficits in different cognitive domains and explored whether these cognitive deficits were associated with the diurnal salivary cortisol levels.

## Methods

### Participants and procedure

Consecutive patients with FM aged 20–60 years were enrolled from the Neurological Institute of Taipei Veterans General Hospital in Taiwan. All patients fulfilled the modified 2010 American College of Rheumatology (ACR) Fibromyalgia Diagnostic Criteria^1^; however, those with any autoimmune rheumatic disease were excluded. Healthy volunteers who did not have past or family histories of FM and who had not experienced any significant pain during the past year were recruited as controls. All participants denied having any history of systemic or major neuropsychiatric disease and exhibited normal results of physical and neurological examinations and brain magnetic resonance imaging (MRI). Participants who were receiving any medication or hormone therapy on a daily basis were excluded. Shift workers and patients who experienced severe dry mouth that may interfere with diurnal cortisol measurement or saliva sampling were also excluded. The institutional review board of Taipei Veterans General Hospital approved the study protocol, and each participant provided written informed consent. All methods were carried out in accordance with relevant guidelines and regulations (Declaration of Helsinki).

At the first visit, all patients with FM and healthy controls completed a questionnaire on the extent of body pain (Widespread Pain Index, [WPI]; range 0–19)^1^, body pain intensity (0–10 on a numerical rating scale), duration (in years) of body pain, and number and severity of the centralized symptoms of chronic pain (Symptom Severity Scale [SSS]; range 0–12), including fatigue, unrefreshing sleep, cognitive symptoms, headache, lower abdominal pain or cramps, and depression^1^. The FIQR was administered to all participants to assess their FM-related functional disability^18^. To evaluate depression severity, the Beck Depression Inventory version 1 (BDI-I) was administered to all participants^25^. Each participant also completed a manual tender point survey. Trained research assistants palpated on the 18 specific anatomical positions defined by the 1990 ACR FM classification at a pressure of 4.0 kg/m^2^ as measured using a dolorimeter^26^. Each participant reported the level of tenderness (0: none; 1: mild; 2: moderate; and 3: severe) at each position. The total tender points (TTP; range 0–18) and total tenderness score (TTS = sum of the tenderness level in the 18 positions; range 0–54) of each participant were determined. After completion of the standardized clinical and questionnaire assessments, all participants were scheduled for another visit to undergo cognitive assessment (see 2.2 and 2.3) and were instructed to collect saliva samples at home for diurnal cortisol measurement on the previous day of visit (see 2.4).

### Subjective cognitive complaint questionnaire

We designed a Chinese questionnaire to characterize subjective cognitive complaints by asking Yes/No questions about difficulties faced by patients in various daily activities within the past 2 years. This questionnaire comprised 12 questions to identify subjective complaints in 4 different cognitive domains. The total score of this questionnaire was the sum of “Yes” to the 12 questions and therefore ranged from 0 to 12. The English version of this questionnaire is provided as Supplementary Table S1. In addition, we used the severity rating of cognitive symptoms in the SSS as a measure of the global function for subjective cognitive complaints.

### Objective cognitive assessments

The objective cognitive assessments were performed by trained psychologists and research assistants, and the results were reviewed and interpreted by psychologists and neurologists. The entire cognitive testing battery included the following tests, which could be categorized into five functional domains:

1. Global function: Mini–Mental State Examination^27^.
2. Memory: Chinese Version Verbal Learning Test (CVVLT)—total correct and 10 min recall (CVVLT–10 M)^28^, Wechsler Memory Scale—logic memory test (WMS–LM), part 1 and 2^29^, and Taylor Complex Figure Test (TY-CFT)—delayed recall parts^30^.
3. Visuospatial: TY-CFT—copy^30^.
4. Language: the modified 30-item Boston Naming Test (BNT)^31^ and Semantic Verbal Fluency Test^32^.
5. Executive: Forward and Backward Digit Span Test^33^, a modification of the Trail-Making Test, part A and B (TMT-A or -B)^34^ and Wisconsin Card Sorting Test—64-card version—total number correct (WCST−TC), perseverative response (WCST–PR), and categories completed^35^.

### Salivary cortisol level measurement

One day prior to the aforementioned cognitive assessments, all participants were instructed to follow the protocol modified from the work of McLean, et al.^36^ to collect their saliva in sample tubes at home at four time points: awakening, 30 min after awakening, 3 pm, and bedtime. For saliva sampling at awakening, participants were asked not to brush their teeth, eat, or drink before sampling. All collected saliva samples were brought to the hospital the next day and preserved in our laboratory refrigerator at − 20 °C until further analysis using commercial enzyme-linked immunosorbent assay kits, AssayMax ELISA Kits (Assaypro, St. Charles, MO, USA). The cortisol awakening response (CAR) was calculated as the difference in cortisol levels between awakening and 30 min after awakening^37^.

### Statistical analysis

Because of the small sample size, Mann–Whitney *U* tests and chi-square tests were used to examine intergroup differences in demographic and clinical profiles, subjective cognitive complaints, and diurnal cortisol levels. For objective cognitive testing, we used stratification (education years ≤ 12 vs. > 12) to adjust education in both FM and control groups. After stratification for education, fewer participants (both FM and controls) were in the lower education group. Stratification was also applied on the results of subjective cognitive complaints questionnaire to compare the subjective and objective cognitive deficits. Correlation analysis was used to explore the association among demographic and clinical profiles, items of the subjective cognitive complaint questionnaire, objective cognitive testing, and diurnal cortisol levels. For the effect size, *φ* was for the between group comparisons of the subjective cognitive complaints questionnaire by chi-square test, Cohen’s *d* for the continuous variables such as total score of the subjective cognitive complaints questionnaire and diurnal cortisol levels, and Hedges’ *g* for the objective cognitive tests after stratification because of smaller sample size. SPSS version 22 (IBM Inc., Armonk, NY, USA) was used to perform the aforementioned statistical analyses, and a *P* value of < 0.05 was considered statistically significant. The effect size was small, medium and large if 0.1, 0.3, 0.5 for *φ* and 0.2, 0.5, 0.8 for Cohen’s *d*^38^ and Hedges’ *g*^39^, respectively.

## Results

### Demographics and clinical profile

In total, 44 patients with FM and 48 healthy controls were enrolled in this cross-sectional study. Age and sex did not differ between the FM and control groups, whereas the years of education were fewer in the FM group than in the control group (*P* = 0.002; Table 1). As expected, the FM group demonstrated higher TTP and TTS on palpation and reported higher scores on the WPI. Moreover, compared with controls, a higher proportion of patients with FM reported fatigue, cognitive symptoms, unrefreshed sleep, headache, and depression on the SSS; the total score on the SSS was also higher in the FM group. The scores on the BDI-I and FIQR were also higher in the FM group than in the control group (Table 1).

**Table 1:** Demographics and clinical data.

	Controls (n = 48)	FM (n = 44)	*P*
Female	38 (79%)	40 (91%)	0.117
Age (year)	50.8 ± 7.2	50.7 ± 6.5	0.969
Education (year)	15.3 ± 2.7	13.9 ± 2.2	**0.002**
Disease duration (year)	–	3.9 ± 1.2	–
Average pain intensity	–	5.9 ± 2.4	–
TTP	4.7 ± 4.2	14.4 ± 3.0	** < 0.001**
TTS	5.9 ± 6.1	26.5 ± 9.3	** < 0.001**
WPI	1.4 ± 2.1	10.3 ± 4.1	** < 0.001**
SSS	2.6 ± 2.0	8.0 ± 2.1	** < 0.001**
Fatigue = 0	22 (50%)	2 (5%)	** < 0.001**
Fatigue = 1	18 (41%)	10 (24%)	
Fatigue = 2	4 (9%)	14 (33%)	
Fatigue = 3	0 (0%)	16 (38%)	
Cognitive symptoms = 0	20 (42%)	3 (7%)	** < 0.001**
Cognitive symptoms = 1	21 (48%)	9 (21%)	
Cognitive symptoms = 2	3 (7%)	21 (50%)	
Cognitive symptoms = 3	0 (0%)	9 (21%)	
Unrefreshed sleep = 0	19 (43%)	0 (0%)	** < 0.001**
Unrefreshed sleep = 1	22 (50%)	5 (12%)	
Unrefreshed sleep = 2	3 (7%)	21 (50%)	
Unrefreshed sleep = 3	0 (0%)	16 (38%)	
Headache = 1	16 (36%)	38 (91%)	** < 0.001**
Abdominal pain = 1	7 (16%)	14 (33%)	0.060
Depression = 1	6 (14%)	25 (60%)	** < 0.001**
BDI−I	5.2 ± 4.8	15.7 ± 9.9	** < 0.001**
FIQR	6.6 ± 8.8	50.9 ± 21.7	** < 0.001**

Data are mean ± SD or number (%).

*FM* fibromyalgia, *TTP* total tender points, *TTS* total tender point score, *WPI* widespread pain index, *SSS* symptom severe score, *BDI–I* Beck's Depression Inventory version 1, *FIQR* Revised fibromyalgia impact questionnaire.

### Subjective cognitive complaint questionnaire

Compared with the control group, the FM group reported more subjective cognitive complaints, mostly with medium effect sizes for each item (Table 2). In the FM group, 90%, 60%, 33%, and 16% of patients reported at least one symptom in executive, memory, language, and visuospatial domains, respectively. The total score on the subjective cognitive complaint questionnaire was worse in the FM group with a large effect size (FM: 4.1 ± 2.6; controls: 1.3 ± 2.2, *P* < 0.001, *d* = 1.171). The total score was highly correlated with the severity of cognitive symptoms in the SSS (0–3) in all participants (FM and controls combined, *r* = 0.537, *P* < 0.001) and controls (*r* = 0.478, *P* = 0.001) but not in the FM group (*r* = 0.117, *P* = 0.485). The total score in the FM group was moderately correlated with functional disability (FIQR, *r* = 0.335, *P* = 0.040). After stratification, patients with FM still reported more subjective cognitive complaints than the controls regardless the education level (Supplementary Table S2 and S3).

**Table 2:** Subjective cognitive complaints questionnaire*.

	Controls (n = 47)		FM (n = 40)	*P*	Effect size
**Memory**					
Q1: Forget where items are located		7 (15%)	19 (48%)	**0.001**	0.355
Q2: Forget the date or the time of an appointment		2 (4%)	6 (15%)	0.084	0.185
Q3: Forget the news you have just seen		3 (6%)	14 (35%)	**0.001**	0.360
Q4: Forget to pay bills		3 (6%)	11 (28%)	**0.008**	0.286
**Visuospatial**					
Q5: Easily go in the wrong direction or get lost		2 (4%)	6 (15%)	0.084	0.185
**Language**					
Q6: Loss for words during conversation		3 (6%)	11 (28%)	**0.008**	0.286
Q7: Difficulty understanding what other people say		2 (4%)	3 (8%)	0.517	0.069
**Executive**					
Q8: Difficulty in counting the total prices		2 (4%)	9 (23%)	**0.011**	0.274
Q9: Easily distracted or concentrating		12 (26%)	24 (60%)	**0.001**	0.349
Q10: Difficulty in learning to use new tools		10 (21%)	23 (58%)	**0.001**	0.372
Q11: Difficulty in making plans you seldom do		4 (9%)	9 (23%)	0.068	0.196
Q12: Irritable when doing somethings unfamiliar		10 (21%)	28 (70%)	** < 0.001**	0.490
Total score		1.3 ± 2.2	4.1 ± 2.6	** < 0.001**	1.171

Data are mean ± SD, number (%) or % (n/N). Effect sizes are expressed as *φ* for Q1–Q12 and Cohen's *d* for the total score.

*FM* fibromyalgia.

*Detailed description of each question is listed in the supplementary table S1.

### Objective cognitive function testing

The raw scores of the objective cognitive assessments of both groups were listed as Table 3. In the lower education subgroup analysis, objective cognitive performance did not differ between FM and control subjects (Table 4). In the higher education subgroup analysis, the results in the FM group performed poorer in memory (CVVLT−10 M, 7.9 ± 1.5 vs. 8.6 ± 0.6, *P* = 0.027, *g* = 0.641; TY−CFT delayed recall, 33.3 ± 2.0 vs. 34.0 ± 1.9, *P* = 0.041, *g* = 0.351), language (BNT: 28.9 ± 1.0 vs. 29.5 ± 1.0, *P* = 0.002, *g* = 0.584), and executive domains (WCST−TC: 42.1 ± 9.9 vs. 47.2 ± 8.2, *P* = 0.026, *g* = 0.026; WCST−PR: 11.8 ± 7.7 vs. 8.0 ± 5.0, *P* = 0.018, *g* = 0.593). The effect sizes were medium with the largest one in the memory domain. Furthermore, several correlations existed between the items of subjective cognitive complaints and objective cognitive testing either in the FM group (Supplementary Table S4) or in all participants (FM and controls combined; Supplementary Table S5). Notably, correlations were observed in language and executive domains but not in the memory and visuospatial domains in the FM group. Also, no correlation was observed between the total score on the subjective cognitive complaint questionnaire and objective cognitive performance.

**Table 3:** Results of objective cognitive tests.

	Controls (n = 45)		FM (n = 43)		*P*	Effect size
MMSE	29.4	± 0.8	28.9	± 1.1	0.009	0.522
**Memory**						
CVVLT−TC	30.5	± 2.8	29.3	± 3.0	0.058	0.414
CVVLT−10 M	8.3	± 1.1	7.8	± 1.5	0.039	0.381
WMS−LM (I)	14.8	± 3.8	13.1	± 4.4	0.095	0.414
WMS−LM (II)	13.6	± 4.4	11.4	± 4.4	0.021	0.500
TY−CFT (delayed recall)	25.5	± 6.1	22.8	± 6.5	0.031	0.429
**Visuospatial**						
TY−CFT (copy)	33.8	± 2.0	33.2	± 1.9	0.081	0.307
**Language**						
BNT	29.4	± 1.0	28.8	± 0.9	< 0.001	0.630
VFT	21.4	± 4.8	18.9	± 5.4	0.011	0.490
**Executive**						
Digit span forward	8.7	± 0.6	8.6	± 0.6	0.796	0.167
Digit span backward	5.8	± 1.3	5.4	± 1.3	0.155	0.308
TMT−A (sec)	9.6	± 4.9	11.0	± 5.8	0.262	0.261
TMT−B (sec)	23.0	± 8.8	28.8	± 15.3	0.093	0.467
WCST−TC	45.6	± 9.8	38.8	± 11.5	0.003	0.638
WCST−PR	9.3	± 6.5	12.9	± 7.2	0.004	0.525
WCST−CC	3.2	± 1.4	2.4	± 1.5	0.013	0.552

All data are raw score (mean ± SD). Effect sizes are expressed as Cohen's *d.*

*FM* fibromyalgia, *MMSE* Mini–Mental State Examination, *CVVLT–TC* Chinese Version Verbal Learning Test, total correct. *CVVLT–10 M* Chinese Version Verbal Learning Test, 10 min recall, *WMS–LM (I or II)* Wechsler Memory Scale—logic memory test, part I or II, *TY–CFT* Taylor Complex Figure Test, *BNT* the modified 30-item Boston Naming Test, *VFT* Categorical Verbal Fluency Test, *TMT* a modification of the Trail-Making Test, *WCST*−*TC* Wisconsin Card Sorting Test—total number correct, * WCST*−*PR* Wisconsin Card Sorting Test—perseverative response. *WCST*−*CC* Wisconsin card sorting test—categories completed.

**Table 4:** Results of objective cognitive tests, stratified according to education.

	Education years ≦ 12						Education years > 12					
	Controls (n = 9)		FM (n = 17)		*P*	Effect size	Controls (n = 39)		FM (n = 27)		*P*	Effect size
MMSE	29.2	± 1.3	28.7	± 1.2	0.111	0.377	29.5	± 0.7	29.0	± 1.0	0.075	0.583
**Memory**												
CVVLT−TC	29.2	± 3.7	28.5	± 2.7	0.302	0.212	30.8	± 2.5	29.8	± 3.1	0.312	0.353
CVVLT−10 M	7.1	± 2.0	7.6	± 1.5	0.559	0.276	8.6	± 0.6	7.9	± 1.5	0.027	0.641
WMS−LM (I)	12.9	± 4.7	11.9	± 4.4	0.627	0.207	15.2	± 3.6	13.8	± 4.3	0.269	0.349
WMS−LM (II)	11.1	± 5.7	10.1	± 4.1	0.464	0.198	14.1	± 4.0	12.2	± 4.5	0.121	0.439
TY−CFT (delayed recall)	21.0	± 6.6	21.2	± 7.0	0.821	0.027	34.0	± 1.9	33.3	± 2.0	0.041	0.351
**Visuospatial**												
TY−CFT (copy)	32.9	± 2.0	32.9	± 1.7	0.870	0.000	26.6	± 5.6	23.8	± 6.1	0.122	0.469
**Language**												
BNT	29.2	± 0.8	28.7	± 0.8	0.155	0.582	29.5	± 1.0	28.9	± 1.0	0.002	0.584
VFT	17.8	± 3.8	17.0	± 5.1	0.498	0.158	22.3	± 4.6	20.1	± 5.4	0.066	0.433
**Executive**												
Digit Span Forward	8.8	± 0.4	8.5	± 0.7	0.288	0.453	8.6	± 0.6	8.7	± 0.5	0.550	0.173
Digit Span Backward	4.9	± 1.6	4.8	± 1.3	0.956	0.066	5.9	± 1.1	5.8	± 1.2	0.552	0.085
TMT−A (sec)	12.0	± 7.1	12.6	± 6.1	0.705	0.087	9.1	± 4.1	10.0	± 5.5	0.647	0.186
TMT−B (sec)	31.3	± 9.5	37.1	± 19.1	0.706	0.326	21.0	± 7.5	23.5	± 9.5	0.422	0.291
WCST−TC	38.1	± 13.4	32.5	± 11.9	0.194	0.420	47.2	± 8.2	42.1	± 9.9	0.026	0.556
WCST−PR	15.5	± 9.2	14.9	± 6.0	0.918	0.077	8.0	± 5.0	11.8	± 7.7	0.018	0.593
WCST−CC	2.3	± 1.6	1.6	± 1.5	0.330	0.425	3.4	± 1.3	2.8	± 1.4	0.080	0.435

All data are raw score (mean ± SD). Effect sizes are expressed as Hedges' g.

*FM* fibromyalgia, *MMSE* Mini–Mental State Examination, *CVVLT–TC* Chinese Version Verbal Learning Test, total correct, *CVVLT–10 M* Chinese Version Verbal Learning Test, 10 min recall, *WMS–LM (I or II)* Wechsler Memory Scale—logic memory test, part I or II, *TY–CFT* Taylor Complex Figure Test, *BNT* the modified 30-item Boston Naming Test, *VFT* Categorical Verbal Fluency Test, *TMT* a modification of the Trail-Making Test, *WCST*−*TC* Wisconsin Card Sorting Test—total number correct, *WCST*−*PR* Wisconsin card sorting test—perseverative response, *WCST*−*CC* Wisconsin card sorting test—categories completed.

### Diurnal cortisol levels and clinical correlation

**Figure 1:**
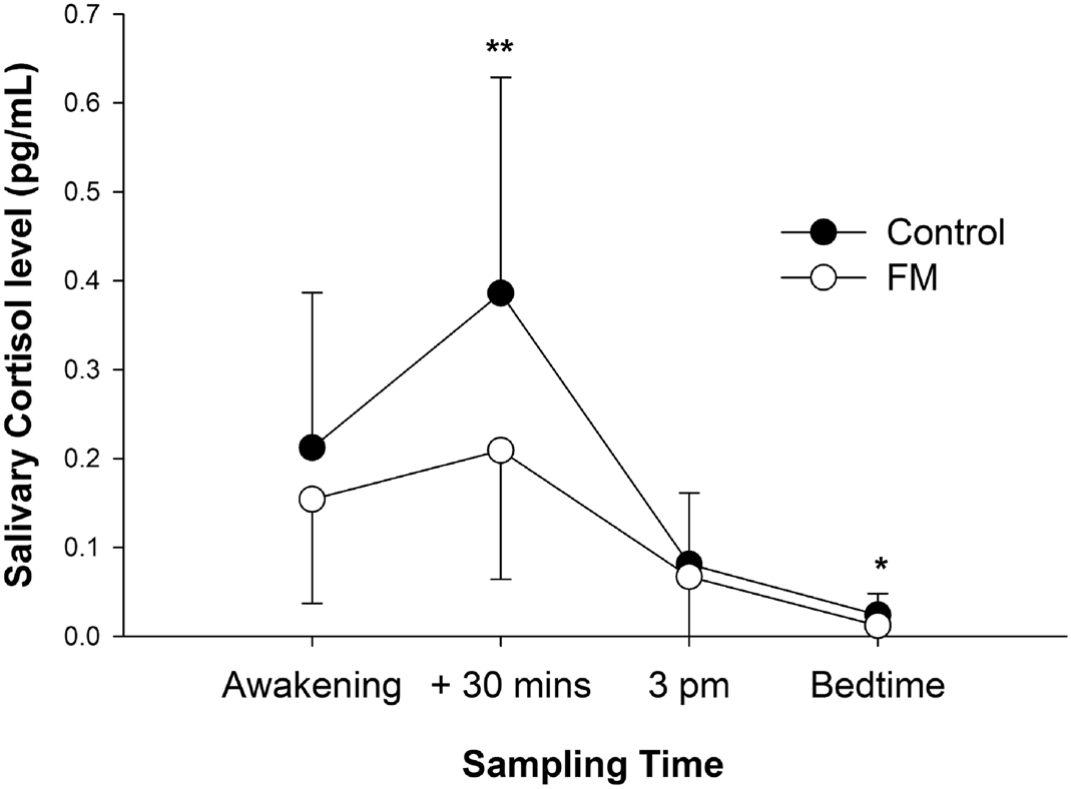
Diurnal salivary cortisol levels. Bars show the mean ± SD; the SD is drawn only in uni-direction. * *P* < 0.05, ** *P* = 0.001.

Diurnal cortisol levels in the FM group tended to be lower than those in the control group (Fig. 1). In particular, the cortisol levels were lower in the FM group at 30 min after awakening with a large effect size (FM: 0.209 ± 0.145, controls: 0.386 ± 0.243 pg/mL, *P* = 0.001, *d* = 0.890) and at bedtime with a medium effect size (FM: 0.002 ± 0.018, controls: 0.024 ± 0.024 pg/mL, *P* = 0.021, *d* = 0.568) but not at awakening (FM: 0.154 ± 0.117, controls: 0.212 ± 0.175 pg/mL, *P* = 0.148, *d* = 0.392) and 3 pm (FM: 0.067 ± 0.067, controls: 0.081 ± 0.080 pg/mL, *P* = 0.503, *d* = 0.190). The CAR was also lower in the FM group with a medium effect size (FM: 0.056 ± 0.138, controls: 0.161 ± 0.185 pg/mL, *P* = 0.011, *d* = 0.646). No correlation was observed between the diurnal cortisol levels and demographics, subjective cognitive complaints, and most clinical profiles (WPI, SSS, and BDI-I) in the FM group. However, the TTP was mild to moderately correlated with the cortisol levels at awakening (*r* =  − 0.253, *P* = 0.029) and 30 min after awakening (*r* =  − 0.341, *P* = 0.003). Correlations between the diurnal cortisol levels and objective cognitive performance were noted in several cognitive domains, such as memory, visuospatial, language and executive functions in the FM group (Supplementary Table S6). In contrast, the correlations were fewer and only observed in the executive domain when all participants (both FM and controls) were included in the correlation analysis (Supplementary Table S7). Among the cognitive tests showing differences between FM and controls, moderate positive correlations existed between the CVVLT−10 M and the cortisol level at 30 min after awakening (*r* = 0.385, *P* = 0.014) and between the BNT and the awakening cortisol level within the FM group (*r* = 0.367, *P* = 0.020). The CAR was not correlated with any objective cognitive tests.

Depression (the BDI-I score) was not correlated with subjective memory complaints or most of the items of the objective cognitive tests in the FM (Supplementary Table S4), and the aforementioned differences between the groups remained the same after adjustment for the BDI-I score.

## Discussion

In this study, we characterized changes in the cognitive function of patients with FM and investigated their relationship with the clinical profile of FM and diurnal cortisol levels. Patients with FM had subjective cognitive complaints regarding executive, language, and memory domains. In objective cognitive testing, patients with FM demonstrated deficits mainly in memory, followed by executive, and language functions. In addition, patients with FM displayed a lower diurnal pattern of cortisol levels, particularly at 30 min after awakening with a large effect size and at bedtime with a moderate effect size. Moderate positive correlations existed between the CVVLT−10 M and the cortisol level at 30 min after awakening and between the BNT and awakening cortisol level within the FM group.

Concerning subjective cognitive complaints, our study demonstrated that patients with FM faced difficulties in memory, executive, and language domains. In agreement with our findings, subjective complaints in memory^40,41^, executive^42^, and language domains^43^ have been reported in patients with FM. A recent review summarizing 52 studies on cognitive function in FM also concluded that memory, attention (executive), and word-finding difficulty (language and semantic memory) are the principal subjective and objective cognitive deficits in these patients^17^. Consistent with this review, the present study also demonstrated deficits in memory (delayed recall in CVVLT and TY−CFT), executive (WCST) and language domains (BNT) in patients with FM.

Previous neuroimaging studies have provided a biological basis for cognitive dysfunction in patients with FM. Specifically, poor performance on WCST in our patients with FM may represent disrupted problem-solving, an ineffective hypothesis-testing strategy^35^, increased distractibility, or rule detection deficit^44^. A pertinent MRI study in FM revealed trophic changes in the anterior cingulated cortex, a brain area related to executive function^45^. The episodic memory deficits in delayed recall of CVVLT and TY−CFT were supported by another MRI study demonstrating decreased bilateral hippocampal volumes in FM^46^. On the other hand, though the BNT is commonly categorized as a language test, the worse BNT in our patients with FM may reflect poor access to semantic memory^47^. Reilly, et al.^48^ demonstrated the utility of the BNT to assess semantic deficits in patients with Alzheimer’s disease and semantic dementia, and several studies also have demonstrated semantic access deficits in patients with FM^49^. Moreover, the semantic access also relies on the function of the hippocampus^50^. Sawrie, et al.^51^ demonstrated that the performance on the BNT is associated with the hippocampus in patients with intractable temporal lobe epilepsy on quantitative ^1^H magnetic resonance spectroscopy. Thus, both episodic and semantic memory deficits may be linked to hippocampal dysfunction.

In addition to neuroimaging, an association between subjective cognitive complaints and FIQR was demonstrated in the present study and the study conducted by Gelonch, et al.^42^. As reported by Williams, et al.^43^, this association may imply that the subjective cognitive dysfunction in patients with FM may additionally result from pain severity, fatigue, mood, sleep, and other insidious clinical factors that interactively affect the overall perception of functional disability. Given the close relationship between these factors and stress, we assumed that stress is an omnibus factor that directly or indirectly affects the cognitive function of patients with FM^2^. In agreement, our data demonstrated that stress, as assessed by salivary cortisol levels, was associated with TTP in all participants and with the performance of patients with FM on the CVVLT–10 M and BNT.

The relationship between the altered cortisol levels and cognitive functions has been observed in several diseases. Studies on PTSD^52^, CFS^17^, Cushing syndrome^53^, and Addison’s disease^22^ have reported deficits mostly in memory, executive function, and attention, in line with our results. However, few studies have investigated the relationship between cortisol and cognition in specific functional domains in patients with FM. Sephton, et al.^54^ demonstrated that the mean diurnal levels of salivary cortisol were positively correlated with the performance on the visual reproduction task. Barcelo-Martinez, et al.^55^ demonstrated a negative correlation of the diurnal difference (the cortisol level at 8 am to 9 am—cortisol level at 4 pm to 5 pm) in serum cortisol levels with verbal memory tested using the Rey Auditory Verbal Learning Test and cognitive flexibility tested using the WCST, suggesting that a more fluctuated diurnal cortisol level may impair the cognitive function. However, in the study by Barcelo-Martinez et al., the cortisol level in the FM group was comparable to but tended to be higher than that in the control group, different from our patient group with lower cortisol levels. This difference might be because of the difference in the sampling method. Note that the stress of venepuncture in the study by Barcelo-Martinez et al. may have also biased the subsequent data on that day^56^.

Our study demonstrated that hypocortisolism is associated with cognitive change. The correlations between WMS–LM, TMT-B and diurnal cortisol levels may imply that attenuated diurnal cortisol levels account for the cognitive deficits. Especially, in the two cognitive tests that showed groups differences, moderate positive correlations were noted between the BNT and the awakening cortisol level, and between the CVVLT–10 M and the cortisol level at 30 min after awakening. Therefore, we suggested a generally inadequate diurnal cortisol level may be harmful to cognitive function in patients with FM. However, not all correlations between cortisol and objective cognitive tests in the FM group showed consistent results. For example, the digital backward showed a negative correlation with the awakening cortisol level. Further studies are needed to clarify the inconsistent cortisol effect upon different cognitive tests.

Although no direct evidence supports the link between altered cortisol levels and both episodic and semantic memory deficits, some indirect evidence might suggest hippocampus as a possible neural substrate for this link. The hippocampus is a limbic structure that is closely related to stress processing^20^ and both episodic and semantic memory function^50^, as tested by the CVVLT and BNT in the present study. Earlier studies in patients with PTSD reported a correlation between hippocampal atrophy and the accumulative effect of chronic stress (disease duration)^57^ and also a correlation between hippocampal atrophy and verbal memory deficits^58^. Taken together, we propose that the memory deficit in patients with FM may be associated with hippocampal dysfunction secondary to chronic stress.

Intriguingly, not all functional domains involved in subjective cognitive complaints from patients with FM demonstrated objective deficits on cognitive function testing. The subjective cognitive complaints were only correlated with the objective cognitive performances in the language and executive domains but not in the memory and visuospatial domains. In the lower education subgroup analysis, the FM group had more subjective cognitive complaints than the controls but comparable objective cognitive performances in memory and executive domains. In fact, such a “mismatch” in cognitive function has been observed in CFS^17^, a common comorbidity of FM, and other clinical pain conditions such as migraine^59^. Biologically, the mismatch may be explicable by cognitive compensation amid objective function testing. In functional MRI studies, subjective illness experience and objective performance revealed separate neural substrates^60^. Another study revealed that patients with FM recruited additional neural networks to achieve the same performance on the Go/NO-go test^61^, and the resultant mental overloading and exhaustion may aggravate subjective cognitive impairment. Psychologically, several potential factors may lead to greater subjective experience of cognitive deficits (compared with objective cognitive testing). In addition to the aforementioned perceived functional disability and mental exhaustion related to brain processing overloading, memory perfectionism, heightened self-monitoring^17^, and altered interoceptive awareness^62^ may contribute to subjective cognitive dysfunction. Studies using personality inventories have been attempting to establish a “pain personality,” including the previous conversion model and the newer vicious cycle model, which refers to the role of problematic stress coping because of the trait of harm avoidance and low self-directedness^63^.

This study has some limitations. First, women comprised 91% of our FM group, and the generalizability of our data is thus limited. Second, the manual tender point survey was not objective, and interpretation of result may be biased by the location of the survey sites, patient and examiner positioning, order of examination, and pressure application technique^64^. However, our purpose was to confirm that these patients with FM also fulfilled the 1990 ACR criteria and provided an additional assessment of tenderness following the work of Hsiao, et al.^65^. Third, our subjective cognitive complaint questionnaire has not been validated. In fact, validated tools for evaluating subjective cognitive decline are still under development^66^. We attempted to use a comprehensive questionnaire as an exploratory tool to measure subjective cognitive complaints, correlations to the cognitive problems of SSS, and objective cognitive performance (Supplementary Tables S4 and S5) in our study, which may support the validity of our subjective cognitive complaint questionnaire. Fourth, we did not assess the effort required for performing objective cognitive tests. Because body pain may affect motivation, Lockhart and Satya-Murti^67^ emphasized the role of inadequate effort, especially in a clinical scenario involving patients with FM, where the base rate of inadequate effort is up to 35%. However, Pidal-Miranda, et al.^41^ suggested that this effect might be small in clinical studies because these study volunteers usually have higher motivation and less compensation-seeking tendencies. Future studies may incorporate performance-validating tests^68^ to explore effort as a significant contributing factor for subjective or objective cognitive functions in FM. Fifth, the cortisol level of participants was tested only for 1 day, and testing for more days would provide more reliable results, considering day-to-day differences in levels^69^. Finally, our exploratory cross-sectional study demonstrated the relation between stress and cognitive change; a longitudinal study is warranted to confirm the causality.

## Conclusion

Our study demonstrated that patients with FM experienced both subjective and objective cognitive changes in memory, executive and language functions. In objective cognitive testing, both episodic and semantic memory functions in patients with FM were correlated with decreased salivary cortisol levels. The link between cognitive change and cortisol suggests stress maladaptation may play some role in the cognitive dysfunction associated with FM. Additional studies must elucidate whether stress management improves cognitive performance in patients with FM.

## Supplementary Information

Supplementary Tables

## Data Availability

The datasets generated and analyzed during the current study are available from the corresponding author on reasonable request.

## Abbreviations

FM: Fibromyalgia
CFS: Chronic fatigue syndrome
PTSD: Post-traumatic stress syndrome
ACR: American College of Rheumatology
TTP: Total tender points
TTS: Total tender point score
WPI: Widespread pain index
SSS: Symptom severe score
BDI-I: Beck’s depression inventory version 1
FIQR: Revised fibromyalgia impact questionnaire
CVVLT: Chinese Version Verbal Learning Test
CVVLT–10 M: Chinese Version Verbal Learning Test—10 min recall
WMS–LM: Wechsler Memory Scale—logic memory
TY-CFT: Taylor complex figure test
BNT: The modified 30-item boston naming test
TMT-A or -B: A modification of the trail-making test, part A or B
WCST: Wisconsin card sorting test
WCST−TC: Wisconsin card sorting test—total correct
WCST−PR: Wisconsin card sorting test—perseverative response
