# Supplementary material for: Salivary cortisol is associated with cognitive changes in patients with fibromyalgia
Source: Sci Rep. 2021 Jan 14;11:1311. doi: 10.1038/s41598-020-79349-0 (PMC7809444; doi:10.1038/s41598-020-79349-0)
Supplement: Supplementary file 1 — Supplementary Tables [file 41598_2020_79349_MOESM1_ESM.docx]

**Salivary cortisol is associated with cognitive changes in patients with fibromyalgia**

Yi-Ju Lin^1,2^, Yu-Chieh Ko^2,3^, Lok-Hi Chow^2,4^, Fu-Jung Hsiao^5^, Hung-Yu Liu^1,2^, Pei-Ning Wang^1,2,5*^, Wei-Ta Chen^1,2,5*^

^1^ Department of Neurological Institute, Taipei Veterans General Hospital, Taipei, Taiwan

^2^ School of Medicine, National Yang-Ming University, Taipei, Taiwan

^3^ Department of Ophthalmology, Taipei Veterans General Hospital, Taipei, Taiwan

^4^ Department of Anesthesiology, Taipei Veterans General Hospital, Taipei, Taiwan

^5^ Brain Research Center, National Yang-Ming University, Taipei, Taiwan

***Co-correspondence to:** Pei-Ning Wang and Wei-Ta Chen

Address: Neurological Institute, Taipei Veterans General Hospital, No. 201, Sec. 2 Shih-Pai Rd, Taipei 112, TAIWAN

TEL: +886-2-28757578

FAX: +886-2-28757386

Email: pnwang@vghtpe.gov.tw (PN Wang); wtchen@vghtpe.gov.tw (WT Chen)

Supplementary Table S1. Subjective cognitive complaints questionnaire.

| Relative to your prior experience, have you experienced any of the following problems **within the past 2 years**? | Yes / No |
| --- | --- |
| Q1: Easily forget where items are located |  |
| Q2: Easily forget the date or the time of an appointment |  |
| Q3: Easily forget the news or TV program you have just seen |  |
| Q4: Easily forget to pay bills |  |
| Q5: Easily go in the wrong direction or get lost |  |
| Q6: Easily find yourself at a loss for words during conversation |  |
| Q7: Difficulty understanding newspapers, books, or what other people say |  |
| Q8: Difficulty in counting the total costs, prices, or charges during shopping |  |
| Q9: Easily distracted or difficulty concentrating |  |
| Q10: Difficulty in learning to use new tools |  |
| Q11: Difficulty in making plans you seldom do (party, travel…etc.) |  |
| Q12: Being anxious or irritable when doing two things at once or when doing something unfamiliar |  |
| Total score |  |

Supplementary Table S2. Subjective cognitive complaints questionnaire* after stratification (Education years ≦ 12)

|  | Controls (n=9) | | FM (n=14) | *P* | Effect size |
| --- | --- | --- | --- | --- | --- |
| **Memory** | |  |  |  |  |
| Q1: Forget where items are located | | 1 (%) | 8 (%) | **0.040** | 0.460 |
| Q2: Forget the date or the time of an appointment | | 1 (%) | 1 (%) | 1.000 | 0.069 |
| Q3: Forget the news you have just seen | | 2 (%) | 5 (%) | 0.657 | 0.143 |
| Q4: Forget to pay bills | | 1 (%) | 4 (%) | 0.611 | 0.207 |
| **Visuospatial** | |  |  |  |  |
| Q5: Easily go in the wrong direction or get lost | | 2 (%) | 4 (%) | 1.000 | 0.071 |
| **Language** | |  |  |  |  |
| Q6: Loss for words during conversation | | 1 (%) | 5 (%) | 0.340 | 0.273 |
| Q7: Difficulty understanding what other people say | | 1 (%) | 1 (%) | 1.000 | 0.069 |
| **Executive** | |  |  |  |  |
| Q8: Difficulty in counting the total prices | | 1 (%) | 3 (%) | 1.000 | 0.133 |
| Q9: Easily distracted or concentrating | | 2 (%) | 9 (%) | 0.089 | 0.411 |
| Q10: Difficulty in learning to use new tools | | 3 (%) | 12 (%) | **0.023** | 0.537 |
| Q11: Difficulty in making plans you seldom do | | 1 (%) | 4 (%) | 0.611 | 0.207 |
| Q12: Irritable when doing somethings unfamiliar | | 4 (%) | 11 (%) | 0.179 | 0.350 |
| Total score | | 2.2 ± 3.9 | 4.8 ± 3.0 | **0.026** | 0.710 |

Data are mean ± SD, number (%) or % (n/N). Effect sizes are expressed as *φ* for Q1-Q12 and Cohen's *d* for the total score.

* Detailed description of each question is listed in the supplementary table S1.

FM: fibromyalgia

Supplementary Table S3. Subjective cognitive complaints questionnaire* after stratification (Education years > 12)

|  | Controls (n=38) | | FM (n=26) | *P* | Effect size |
| --- | --- | --- | --- | --- | --- |
| **Memory** | |  |  |  |  |
| Q1: Forget where items are located | | 6 (%) | 11 (%) | **0.018** | 0.295 |
| Q2: Forget the date or the time of an appointment | | 1 (%) | 5 (%) | **0.036** | 0.280 |
| Q3: Forget the news you have just seen | | 1 (%) | 9 (%) | **0.001** | 0.433 |
| Q4: Forget to pay bills | | 2 (%) | 7 (%) | **0.025** | 0.306 |
| **Visuospatial** | |  |  |  |  |
| Q5: Easily go in the wrong direction or get lost | | 0 (%) | 2 (%) | 0.161 | 0.217 |
| **Language** | |  |  |  |  |
| Q6: Loss for words during conversation | | 2 (%) | 6 (%) | 0.054 | 0.265 |
| Q7: Difficulty understanding what other people say | | 1 (%) | 2 (%) | 0.561 | 0.118 |
| **Executive** | |  |  |  |  |
| Q8: Difficulty in counting the total prices | | 1 (%) | 6 (%) | **0.015** | 0.322 |
| Q9: Easily distracted or concentrating | | 10 (%) | 15 (%) | **0.012** | 0.316 |
| Q10: Difficulty in learning to use new tools | | 7 (%) | 11 (%) | **0.037** | 0.261 |
| Q11: Difficulty in making plans you seldom do | | 3 (%) | 5 (%) | 0.253 | 0.168 |
| Q12: Irritable when doing somethings unfamiliar | | 6 (%) | 17 (%) | **<0.001** | 0.508 |
| Total score | | 1.1 ± 1.5 | 3.7 ± 2.3 | **<0.001** | 1.356 |

Data are mean ± SD, number (%) or % (n/N). Effect sizes are expressed as *φ* for Q1-Q12 and Cohen's *d* for the total score.

* Detailed description of each question is listed in the supplementary table S1.

FM: fibromyalgia

Supplementary Table S4. Correlational analysis of subjective cognitive complaint questionnaire† and objective cognitive tests in FM group (n=44).

|  | Memory | | | | Visual | Language | | Executive | | | | | **Total**  **score** |
| --- | --- | --- | --- | --- | --- | --- | --- | --- | --- | --- | --- | --- | --- |
|  | Q1 | Q2 | Q3 | Q4 | Q5 | Q6 | Q7 | Q8 | Q9 | Q10 | Q11 | Q12 |  |
| MMSE | .03 | -.07 | -.22 | .02 | .17 | .02 | .01 | -.05 | .15 | -.25 | -.05 | .35* | .01 |
| **Memory** |  |  |  |  |  |  |  |  |  |  |  |  |  |
| CVVLT−TC | -.03 | .00 | -.20 | .26 | -.16 | -.44** | -.11 | -.21 | -.21 | -.07 | .25 | -.21 | -.19 |
| CVVLT−10M | -.20 | -.10 | -.17 | .00 | -.25 | -.40* | .10 | -.10 | -.07 | .04 | .15 | -.16 | -.21 |
| WMS−LM (I) | .02 | .18 | .03 | .27 | .00 | -.25 | -.15 | -.08 | -.28 | -.05 | -.16 | -.07 | -.09 |
| WMS−LM (II) | -.01 | .18 | -.02 | .25 | -.02 | -.36* | -.08 | -.01 | -.29 | -.11 | -.04 | -.02 | -.10 |
| TY−CFT (delayed) | .01 | -.22 | -.19 | .18 | -.22 | -.17 | .12 | -.22 | -.10 | -.13 | -.09 | -.14 | -.20 |
| **Visuospatial** |  |  |  |  |  |  |  |  |  |  |  |  |  |
| TY−CFT (copy) | .29 | -.07 | .15 | .08 | .01 | -.01 | -.07 | .00 | .14 | .11 | .06 | .22 | .18 |
| **Language** |  |  |  |  |  |  |  |  |  |  |  |  |  |
| BNT | .12 | .30 | -.20 | .23 | .15 | -.30 | .05 | .16 | .01 | .00 | .16 | -.01 | .10 |
| VFT | -.12 | .02 | -.23 | .17 | -.15 | -.38* | .07 | .02 | -.17 | -.16 | .12 | -.17 | -.19 |
| **Executive** |  |  |  |  |  |  |  |  |  |  |  |  |  |
| Digital forward | -.03 | -.03 | -.08 | .03 | -.03 | -.17 | -.36* | -.14 | -.35* | -.19 | -.14 | -.06 | -.25 |
| Digital backward | .17 | -.01 | .03 | .29 | -.13 | -.22 | -.05 | -.19 | -.24 | -.21 | -.23 | .01 | -.13 |
| TMT−A (sec) | -.21 | -.17 | -.04 | -.08 | -.22 | .11 | .09 | -.28 | -.22 | .03 | -.29 | -.16 | -.25 |
| TMT−B (sec) | -.18 | -.12 | -.04 | -.07 | .00 | .28 | -.06 | -.03 | -.19 | .11 | -.16 | -.10 | -.09 |
| WCST−TC | -.16 | .07 | -.04 | -.02 | .10 | .08 | -.26 | -.13 | -.04 | -.18 | -.24 | .01 | -.13 |
| WCST−PR | .02 | -.10 | .08 | .06 | .00 | .17 | .12 | .01 | -.05 | -.15 | -.07 | .05 | .02 |
| WCST−CC | -.09 | .12 | -.06 | -.01 | .12 | .06 | -.26 | -.10 | -.09 | -.21 | -.22 | .03 | -.12 |

Data present as correlation coefficient. * *P* < 0.05, ** *P* < 0.01.

† Detailed description of each question in the subjective cognitive complaint questionnaire is listed in the Supplementary Table S1.

MMSE: Mini–Mental State Examination. CVVLT−TC: Chinese Version Verbal Learning Test, total correct. CVVLT−10M: Chinese Version Verbal Learning Test, 10 minutes recall. WMS–LM (I or II): Wechsler Memory Scale – logic memory test, part I or II. TY–CFT: Taylor Complex Figure Test. BNT: the modified 30-item Boston Naming Test. VFT: Semantic Verbal Fluency Test. TMT−A or −B: a modification of Trail-Making Test, part A or B. WCST−TC: Wisconsin Card Sorting Test – total number correct. WCST−PR: Wisconsin Card Sorting Test – perseverative response. WCST−CC: Wisconsin Card Sorting Test – categories completed

Supplementary Table S5. Correlational analysis of subjective cognitive complaint questionnaire† and objective cognitive tests in all participants (n=92).

|  | Memory | | | | Visual | Language | | Executive | | | | | **Total**  **score** |
| --- | --- | --- | --- | --- | --- | --- | --- | --- | --- | --- | --- | --- | --- |
|  | Q1 | Q2 | Q3 | Q4 | Q5 | Q6 | Q7 | Q8 | Q9 | Q10 | Q11 | Q12 |  |
| MMSE | -.13 | -.21* | -.35** | -.14 | -.12 | -.18 | -.17 | -.21 | -.07 | -.29** | -.17 | -.05 | -.29** |
| **Memory** |  |  |  |  |  |  |  |  |  |  |  |  |  |
| CVVLT−TC | -.15 | -.18 | -.31** | .00 | -.247* | -.45** | -.27* | -.26* | -.25* | -.15 | .02 | -.25* | -.35** |
| CVVLT−10M | -.19 | -.23* | -.30** | -.14 | -.32** | -.41** | -.13 | -.23* | -.12 | -.11 | -.05 | -.23* | -.34** |
| WMS−LM (I) | -.11 | .03 | -.19 | .08 | -.19 | -.28** | -.24* | -.17 | -.22* | -.09 | -.12 | -.06 | -.21* |
| WMS−LM (II) | -.13 | .01 | -.28** | .04 | -.24* | -.36** | -.17 | -.14 | -.25* | -.17 | -.05 | -.06 | -.25* |
| TY−CFT (delayed) | -.13 | -.29** | -.26* | -.04 | -.29** | -.29** | -.09 | -.27* | -.13 | -.17 | -.11 | -.22* | -.31** |
| **Visuospatial** |  |  |  |  |  |  |  |  |  |  |  |  |  |
| TY−CFT (copy) | .06 | -.14 | .01 | -.06 | -.08 | -.11 | -.06 | -.06 | -.01 | -.06 | .00 | -.06 | -.07 |
| **Language** |  |  |  |  |  |  |  |  |  |  |  |  |  |
| BNT | -.02 | .07 | -.31** | .03 | -.09 | -.25* | -.04 | -.02 | -.10 | -.12 | .03 | -.22* | -.16 |
| VFT | -.11 | -.07 | -.28** | .04 | -.22* | -.38** | -.11 | -.08 | -.11 | -.10 | .06 | -.23* | -.23* |
| **Executive** |  |  |  |  |  |  |  |  |  |  |  |  |  |
| Digital forward | .05 | .03 | .01 | .07 | .03 | -.09 | -.21 | -.04 | -.16 | -.03 | .00 | -.05 | -.05 |
| Digital backward | -.01 | -.13 | -.09 | .08 | -.13 | -.20 | -.09 | -.16 | -.05 | -.13 | -.13 | -.07 | -.15 |
| TMT−A (sec) | .02 | .02 | .12 | .04 | .04 | .23* | .26* | -.06 | .02 | .13 | -.11 | .02 | .09 |
| TMT−B (sec) | .02 | .04 | .13 | .04 | .17 | .37** | .13 | .08 | .01 | .24* | -.04 | .09 | .18 |
| WCST−TC | -.17 | -.05 | -.13 | -.10 | .01 | -.05 | -.16 | -.15 | -.08 | -.16 | -.16 | -.14 | -.19 |
| WCST−PR | .12 | .04 | .14 | .14 | .06 | .21 | .10 | .10 | -.02 | .04 | .03 | .20 | .16 |
| WCST−CC | -.11 | -.02 | -.15 | -.10 | .01 | -.05 | -.17 | -.14 | -.13 | -.16 | -.14 | -.13 | -.18 |

Data present as correlation coefficient. * *P* < 0.05, ** *P* < 0.01.

† Detailed description of each question in the subjective cognitive complaint questionnaire is listed in the Supplementary Table S1.

MMSE: Mini–Mental State Examination. CVVLT−TC: Chinese Version Verbal Learning Test, total correct. CVVLT−10M: Chinese Version Verbal Learning Test, 10 minutes recall. WMS–LM (I or II): Wechsler Memory Scale – logic memory test, part I or II. TY–CFT: Taylor Complex Figure Test. BNT: the modified 30-item Boston Naming Test. VFT: Semantic Verbal Fluency Test. TMT−A or −B: a modification of Trail-Making Test, part A or B. WCST−TC: Wisconsin Card Sorting Test – total number correct. WCST−PR: Wisconsin Card Sorting Test – perseverative response. WCST−CC: Wisconsin Card Sorting Test – categories completed

Supplementary Table S6. Correlational analysis of clinical variables of FM and diurnal cortisol levels with objective cognitive tests and total score of subjective cognitive complaint questionnaire in FM group (n=44).

|  |  |  |  |  |  |  | Cortisol levels | | | | |
| --- | --- | --- | --- | --- | --- | --- | --- | --- | --- | --- | --- |
|  | TTP | TTS | WPI | SSS | FIQR | BDI-I | Awakening | 30 mins | 3pm | Bedtime | CAR |
| MMSE | -.20 | -.15 | .02 | -.21 | -.15 | -.19 | .10 | -.11 | .06 | -.13 | -.20 |
| **Memory** |  |  |  |  |  |  |  |  |  |  |  |
| CVVLT−TC | -.01 | .15 | .06 | .19 | -.05 | -.02 | .05 | .18 | .06 | -.09 | .14 |
| CVVLT−10M | -.08 | .03 | .19 | .19 | .10 | -.02 | .22 | .39* | .14 | .10 | .21 |
| WMS−LM (I) | -.12 | -.14 | -.04 | .18 | -.13 | -.12 | -.03 | .11 | .34* | -.37* | .14 |
| WMS−LM (II) | -.18 | -.14 | -.03 | .22 | -.07 | -.13 | .10 | .17 | .40* | -.25 | .10 |
| TY−CFT (delayed) | -.14 | -.13 | .11 | .26 | -.11 | -.10 | -.04 | .13 | .13 | -.23 | .16 |
| **Visuospatial** |  |  |  |  |  |  |  |  |  |  |  |
| TY−CFT (copy) | -.19 | -.02 | .03 | .06 | -.11 | .04 | -.22 | -.38* | -.05 | -.10 | -.21 |
| **Language** |  |  |  |  |  |  |  |  |  |  |  |
| BNT | -.02 | -.11 | .05 | .18 | .10 | -.05 | .37* | .15 | -.02 | -.09 | -.15 |
| VFT | -.11 | -.13 | .06 | .22 | .09 | -.17 | .09 | .06 | .14 | .04 | -.02 |
| **Executive** |  |  |  |  |  |  |  |  |  |  |  |
| Digital forward | -.15 | -.24 | -.13 | -.07 | -.43** | -.35* | .00 | -.02 | .03 | -.15 | -.01 |
| Digital backward | -.11 | -.18 | -.03 | .04 | -.10 | .01 | -.43** | -.13 | -.22 | -.25 | .23 |
| TMT−A (sec) | .06 | .18 | .17 | -.27 | .08 | .13 | -.35* | -.26 | -.15 | .05 | .02 |
| TMT−B (sec) | -.08 | .11 | .08 | -.16 | .05 | .13 | -.29 | -.34* | -.02 | .34* | -.11 |
| WCST−TC | -.15 | -.08 | -.11 | -.03 | -.04 | -.22 | -.09 | -.13 | -.02 | -.02 | -.06 |
| WCST−PR | .17 | .01 | -.06 | -.09 | .00 | .05 | .10 | -.05 | -.11 | -.09 | -.14 |
| WCST−CC | -.18 | -.11 | -.18 | -.05 | -.04 | -.29 | -.04 | -.13 | .04 | .05 | -.10 |
| Total score of subjective  cognitive complaint questionnaire | .13 | .13 | -.10 | .05 | .34* | .21 | .05 | .09 | -.15 | .16 | .05 |

Data present as correlation coefficient. * *P* < 0.05, ** *P* < 0.01*.*

FM: fibromyalgia. TTP: total tender points. TTS: total tender point score. WPI: widespread pain index. BDI–I: Beck's Depression Inventory version 1. FIQR: Revised fibromyalgia impact questionnaire. MMSE: Mini–Mental State Examination. CVVLT−TC: Chinese Version Verbal Learning Test, total correct. CVVLT−10M: Chinese Version Verbal Learning Test, 10 minutes recall. WMS–LM (I or II): Wechsler Memory Scale – logic memory test, part I or II. TY–CFT: Taylor Complex Figure Test. BNT: the modified 30-item Boston Naming Test. VFT: Semantic Verbal Fluency Test. TMT−A or −B: a modification of Trail-Making Test, part A or B. WCST−TC: Wisconsin Card Sorting Test – total number correct. WCST−PR: Wisconsin Card Sorting Test – perseverative response. WCST−CC: Wisconsin Card Sorting Test – categories completed

Supplementary Table S7. Correlational analysis of clinical variables of FM and diurnal cortisol levels with objective cognitive tests and total score of subjective cognitive complaint questionnaire in all participants (n=92).

|  |  |  |  |  |  |  | Cortisol levels | | | | |
| --- | --- | --- | --- | --- | --- | --- | --- | --- | --- | --- | --- |
|  | TTP | TTS | WPI | SSS | FIQR | BDI-I | Awakening | 30 mins | 3pm | Bedtime | CAR |
| MMSE | -.28* | -.34** | -.24* | -.25* | -.34** | -.26* | .03 | .03 | .15 | .15 | -.03 |
| **Memory** |  |  |  |  |  |  |  |  |  |  |  |
| CVLT−TC | -.20 | -.25* | -.15 | -.12 | -.23 | -.22* | .08 | .10 | .11 | .12 | -.01 |
| CVLT−10M | -.19 | -.26* | -.11 | -.12 | -.17 | -.20 | .18 | .21 | .17 | .16 | .08 |
| WMS−LM (I) | -.32** | -.31** | -.24* | -.13 | -.24* | -.19 | -.02 | .03 | .09 | -.10 | .06 |
| WMS−LM (II) | -.34** | -.37** | -.27* | -.13 | -.24* | -.19 | .08 | .10 | .13 | -.02 | .06 |
| TY−CFT (delayed) | -.20 | -.25* | -.16 | -.15 | -.32** | -.21* | -.02 | .10 | .13 | -.03 | .13 |
| **Visuospatial** |  |  |  |  |  |  |  |  |  |  |  |
| TY−CFT (copy) | -.04 | -.16 | -.18 | -.16 | -.27* | -.15 | -.06 | -.04 | .12 | .08 | -.04 |
| **Language** |  |  |  |  |  |  |  |  |  |  |  |
| BNT | -.26* | -.25* | -.28** | -.21 | -.14 | -.18 | .14 | .18 | .06 | .11 | .08 |
| VFT | -.30** | -.30** | -.25* | -.19 | -.14 | -.24* | .18 | .11 | .08 | .12 | -.05 |
| **Executive** |  |  |  |  |  |  |  |  |  |  |  |
| Digital forward | -.03 | -.04 | -.12 | -.07 | -.18 | -.20 | .12 | .13 | .18 | .03 | .04 |
| Digital backward | -.13 | -.16 | -.10 | -.08 | -.17 | -.09 | -.25* | -.02 | -.15 | -.06 | .15 |
| TMT−A (sec) | .23* | .18 | .23* | .08 | .15 | .20 | -.23* | -.20 | -.02 | -.01 | -.05 |
| TMT−B (sec) | .28* | .22* | .28* | .16 | .23 | .24* | -.22 | -.32** | .03 | .15 | -.18 |
| WCST−TC | -.19 | -.28* | -.26* | -.21 | -.30* | -.34** | .04 | .15 | .13 | .14 | .11 |
| WCST−PR | .16 | .27* | .17 | .15 | .27* | .26* | .04 | -.11 | -.15 | -.14 | -.14 |
| WCST−CC | -.18 | -.27* | -.30** | -.21 | -.28* | -.35** | .07 | .18 | .19 | .13 | .15 |
| Total score of subjective  cognitive complaints questionnaire | .61** | .59** | .50** | .59** | .65** | .53** | -.13 | -.26* | -.15 | -.09 | -.19 |

Data present as correlation coefficient. * *P* < 0.05, ** *P* < 0.01*.*

FM: fibromyalgia. TTP: total tender points. TTS: total tender point score. WPI: widespread pain index. BDI–I: Beck's Depression Inventory version 1. FIQR: Revised fibromyalgia impact questionnaire. MMSE: Mini–Mental State Examination. CVVLT−TC: Chinese Version Verbal Learning Test, total correct. CVVLT−10M: Chinese Version Verbal Learning Test, 10 minutes recall. WMS–LM (I or II): Wechsler Memory Scale – logic memory test, part I or II. TY–CFT: Taylor Complex Figure Test. BNT: the modified 30-item Boston Naming Test. VFT: Semantic Verbal Fluency Test. TMT−A or −B: a modification of Trail-Making Test, part A or B. WCST−TC: Wisconsin Card Sorting Test – total number correct. WCST−PR: Wisconsin Card Sorting Test – perseverative response. WCST−CC: Wisconsin Card Sorting Test – categories completed
